# Persistent glycemic deterioration after the COVID-19 pandemic: A large cohort analysis stratifying by care settings, specialties, and gender disparities in Chengdu, China

**DOI:** 10.1016/j.pmedr.2026.103378

**Published:** 2026-01-06

**Authors:** Dongmei Chen, Shiyu Deng

**Affiliations:** a Department of Health Management/International Medical Service, West China School of Medicine, Sichuan University, China; b Sichuan University Affiliated Chengdu Second People's Hospital, Chengdu, China; c Department of Laboratory, West China School of Medicine, Sichuan University, China

**Keywords:** COVID-19, Glycemic control, HbA1c, Healthcare settings, Clinical specialties,Gender disparities

## Abstract

**Objective:**

This study aimed to quantify the sustained impact of the Coronavirus Disease 2019(COVID-19) pandemic on glycemic control across distinct healthcare settings, medical specialties, and demographic groups.

**Methods:**

We conducted a retrospective analysis of 274,909 glycated hemoglobin(HbA1c) records from a tertiary hospital in Chengdu,China between 2013 and 2025. Non-parametric tests were used to compare median HbA1c levels across pre-pandemic, pandemic, peak-infection, and post-pandemic phases, stratified by Healthcare Settings, specialty, age, and gender.

**Results:**

Significant deterioration in glycemic control was observed during the pandemic period and sustained thereafter. Median HbA1c increased from 5.9 % (pre-pandemic) to 6.4 % (peak infection), remaining elevated at 6.2 % post-pandemic (*p* < 0.01). Outpatient settings demonstrated the poorest glycemic control (median 6.4 %). The most pronounced deteriorations occurred in critical care unit (ICU: Δ + 3.0 %) and surgical departments (Δ + 0.7 %), while Endocrinology maintained better outcomes (Δ-0.3 %). Older adults (≥76 years, Δ + 0.6 %) and male patients showed greatest vulnerability to glycemic deterioration.

**Conclusion:**

The COVID-19 pandemic resulted in persistent glycemic deterioration, particularly affecting outpatient management, acute care settings, and vulnerable demographic groups. These findings highlight critical gaps in diabetes care resilience and underscore the need for targeted interventions in high-risk settings and strengthened support systems for susceptible populations.

## Highlights

• Persistent post-pandemic deterioration in glycemic control was observed.
• Outpatient settings demonstrated the poorest glycemic control during the pandemic.
• Acute and critical care settings experienced the most severe glycemic deterioration.
• Specialized diabetes care demonstrated resilient glycemic outcomes.
• Older adults and male patients were most vulnerable to glycemic deterioration.

## 1 Introduction

The collateral damage of pandemic containment measures on chronic disease management has emerged as a major public health concern. While acute metabolic impacts of SARS-CoV-2 infection are well-documented (Yonekawa and Shimono, 2022; Faruqi and Balasubramanyam, 2023; Modes et al., 2022), the systemic effects of healthcare disruptions remain poorly quantified, particularly in low- and middle-income countries (Hogan et al., 2020). Existing studies have three critical limitations: Most analyzed only lockdown periods without post-pandemic recovery data (Izzi-Engbeaya et al., 2021); Few stratified patients by care delivery pathways (Khunti et al., 2022); None accounted for China's unique transition from “Zero-COVID” to reopening (Su et al., 2022). Our study addresses these gaps through a care-continuum lens, providing actionable insights for health system recovery with special emphasis on gender disparities and healthcare-setting variations.

## 2 Methods

### 2.1 Study design and population

This retrospective cohort analysis utilized de-identified electronic health records from a 2600-bed tertiary hospital in Chengdu, China, which serves over 3 million people annually. Data were extracted for the period from January 2013(the inception of the hospital's comprehensive Laboratory Information System) to May 2025. We enrolled participants in the study according to the following criteria: 1) having at least one valid glycated hemoglobin (HbA1c) measurement during the study period, and 2) having a documented visit purpose (e.g., outpatient, inpatient, emergency, or screening). We excluded records with missing or clinically implausible HbA1c values (less than 3.60 % or greater than 20.00 %), laboratory quality control records, data from research protocols, and records with incomplete demographic characteristics.

### 2.2 Measures

#### 2.2.1 Definition of pandemic phases

Pandemic phases were defined based on the biological lag of HbA1c reflection (approximately 2–3 months), combined with China's pandemic response timeline and national seroprevalence data (Zeng et al., 2023), as follows:

**Pre-pandemic period**: January 2013–December 2019.

**Pandemic period**: January 2020–December 2022.

**Peak infection period**: January 2023–March 2023 (about 80 % population seroprevalence).

**Post-pandemic period**: April 2023–May 2025.

#### 2.2.2 Glycemic control outcome

The primary outcome was glycemic control, measured by the HbA1c value (%) extracted from the Laboratory Information System. All HbA1c measurements were included per participant per encounter.

#### 2.2.3 Stratification variables

**Healthcare Settings:**Visits were categorized as inpatient, outpatient, emergency, or screening based on the recorded visit purpose.

**Clinical Departments:** Patient encounters were linked to their primary treating department, including Endocrinology, Cardiology, Neurology, Nephrology, Surgical Departments, Emergency Medicine, Intensive Care Unit (ICU), and Health Management.

**Demographic Variables:** Patient age and gender were extracted from the electronic health records. Age was analyzed both as a continuous variable and categorized into groups (0–18, 19–45, 46–60, 61–75, and ≥ 76 years).

#### 2.2.4 Other variables

Basic demographic information and visit types were used to describe the cohort characteristics across the different pandemic phases.

### 2.3 Statistical analysis

Non-parametric tests (Kruskal-Wallis, Mann-Whitney U) were employed due to non-normal HbA1c distribution. Post-hoc Dunn's tests with Bonferroni adjustment addressed multiple comparisons. Effect sizes were calculated using Cliff's Delta and Epsilon-squared. Statistical significance was set at *p* < 0.05. All analyses were performed using IBM SPSS Statistics 27.0.1.

### 2.4 Ethical considerations

The study was approved by the Institutional Review Board (Approval #[KY]PJ2025416). Waiver of informed consent was granted due to the retrospective nature of the study.

## 3 Results

### 3.1 Data extraction and cleaning

A total of 275,465 records were initially extracted from the Laboratory Information System. After applying exclusion criteria:

188 quality control records;

74 research protocol records;

245 records with missing HbA1c values;

47 records with HbA1c = 0;

2 records with HbA1c ≥100.

The final analytical dataset comprised 274,909 valid records (99.8 % of original data).

### 3.2 Participant characteristics

The final cohort included 274,909 records. Significant shifts in demographics and healthcare utilization were observed across pandemic phases (Table 1). The mean age decreased from 63.19 ± 15.36 years (pre-pandemic) to 58.13 ± 16.67 years (post-pandemic) (*p* < 0.01), suggesting increased engagement from younger populations. The proportion of inpatient visits dominated pre-pandemic (80.5 %) but sharply decreased to 37.5 % post-pandemic, while outpatient visits tripled from 11.2 % to 36.6 %. The proportion of patients with high HbA1c (≥ 6.5 %) surged to 47.9 % during the peak infection period.

**Table 1:** Characteristics of study participants with HbA1c measurements at a tertiary hospital in Chengdu, China, stratified by pandemic phase (2013–2025) (mean ± SD / n (%)).

Characteristic	Pre-pandemic (*n* = 111,149)	Pandemic (*n* = 86,703)	Peak Infection period (*n* = 5974)	Post-pandemic (*n* = 71,083)	Total (*N* = 274,909)
Age (years)	63.2 ± 15.4	58.8 ± 16.8	61.5 ± 15.7	58.1 ± 16.7	60.5 ± 16.3
Female	55,716 (50.1 %)	41,446(47.8 %)	2931(49.1 %)	33,133(46.6 %)	133,206(48.5 %)
Healthcare Settings					
Inpatient	89,428(80.5 %)	42,990(49.6 %)	3454(57.8 %)	26,669(37.5 %)	162,541(59.1 %)
Outpatient	12,527(11.3 %)	16,553(19.1 %)	2106(35.3 %)	26,011(36.6 %)	57,197(20.8 %)
Emergency	334(0.3 %)	198(0.2 %)	22(0.4 %)	353(0.5 %)	907(0.3 %)
screening	8860(8.0 %)	26,962(31.1 %)	392(6.6 %)	18,050(25.4 %)	54,264(19.7 %)
Departments Included in Analysis					
Endocrinology	15,272(13.7 %)	7615(8.8 %)	909(15.2 %)	17,970(25.3 %)	41,766(15.2 %)
Cardiology	13,487(12.1 %)	10,647(12.3 %)	999(16.7 %)	8027(11.3 %)	33,160(12.1 %)
Neurology	16,920(15.2 %)	9625(11.1 %)	416(7.0 %)	4731(6.7 %)	31,692(11.5 %)
Nephrology	2747(2.5 %)	2731(3.1 %)	196(3.3 %)	1866(2.6 %)	7540(2.7 %)
Surgical Department	16,018(14.4 %)	5887(6.8 %)	617(10.3 %)	3998(5.6 %)	26,520(9.6 %)
Emergency Medicine	334(0.3 %)	198(0.2 %)	22(0.4 %)	353(0.5 %)	907(0.3 %)
Intensive Care Unit	1171(1.1 %)	381(0.4 %)	13(0.2 %)	215(0.3 %)	1780(0.6 %)
Health Management	8860(8.0 %)	26,962(31.1 %)	392(6.6 %)	18,050 (25.4 %)	54,264 (19.7 %)
Age(years) Groups					
Youth(<18)	181(0.2 %)	183(0.2 %)	10(0.2 %)	195(0.3 %)	569(0.2 %)
Young-adults [18,39]	8782(7.9 %)	13,598(15.7 %)	619(10.4 %)	11,760(16.5 %)	34,759(12.6 %)
Middle-aged-adults[40,59]	32,169(28.9 %)	28,771(33.2 %)	1966(32.9 %)	23,480(33.0 %)	86,386(31.4 %)
Older-adults (≥60)	70,017(63.0 %)	44,151(50.9 %)	3379(56.6 %)	35,648(50.1 %)	153,195(55.7 %)

Note: SD = Standard Deviation.

### 3.3 Overall trajectory of glycemic control across pandemic phases

**Fig. 1:**
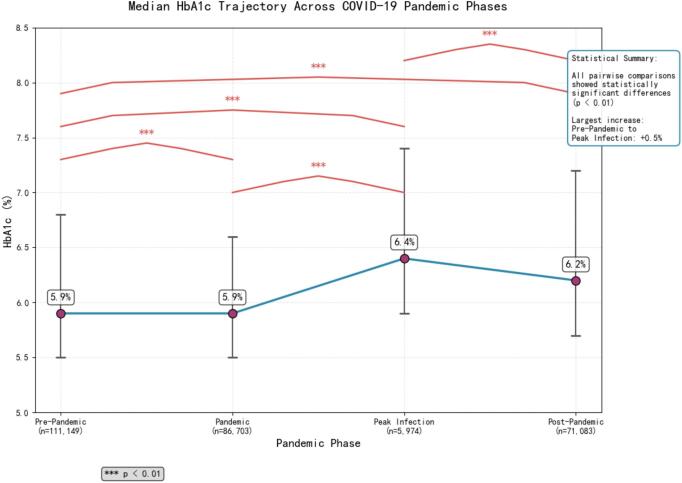
Trajectory of median HbA1c across Coronavirus Disease 2019 (COVID-19) pandemic phases in a cohort of patients from a tertiary hospital in Chengdu, China (2013–2025).

The median HbA1c significantly increased from 5.9 % (interquartile range (IQR): 5.5, 6.8) pre-pandemic to 6.4 % (IQR: 5.9, 7.4) during the peak infection period (*p* < 0.01) and remained elevated at 6.2 % (IQR: 5.7, 7.2) in the post-pandemic period, indicating a sustained deterioration in glycemic control (see Fig. 1).

### 3.4 Disparities in glycemic control by healthcare settings

Glycemic control varied significantly by visit type (Kruskal-Wallis H = 22,875.43, *p* < 0.01). Screening visits demonstrated the best control (median: 5.6 %, IQR: 5.4, 6.0 %). Emergency (6.1 %, IQR: 5.6, 7.0 %) and inpatient visits (6.0 %, IQR: 5.6, 7.0 %) showed intermediate control. Outpatient settings showed the poorest control (median: 6.4 %, IQR: 5.8, 7.5 %; *p* < 0.01 vs. all other types). Stratification by pandemic phase (Table 2) revealed that emergency and inpatient settings experienced the most significant acute deterioration during the peak infection period (Δmedian HbA1c: + 0.7 % and + 0.5 %, respectively), while outpatient control was poor throughout but relatively stable. Screening populations maintained optimal and stable control.

### 3.5 Age and gender differences in glycemic vulnerability

The pandemic's impact on HbA1c levels exhibited a clear gender disparity and age gradient(Table 3).The geriatric population (≥ 76 years) exhibited dual vulnerability, demonstrating both the highest absolute HbA1c levels during the peak period (6.6 % in males, 6.5 % in females) and the greatest magnitude of increase from pre-pandemic levels.

**Table 3:** :HbA1c levels by age group, gender, and pandemic phase in the study cohort from Chengdu, China (2013–2025)(Median[IQR]).

Age group	gender	N	Pre-Pandemic	Pandemic	Peak Infection period	Post-Pandemic	Mean Difference*
0-18y	Male	318	5.4[5.2, 5.6]	5.5[5.4, 5.9]	5.5[5.5, 7.1]	5.6[5.4, 5.9]	+ 0.1
0-18y	Female	251	5.4[5.2, 5.6]	5.5[5.3, 6.1]	5.5[5.5, 8.8]	5.7[5.4, 7.8]	+ 0.1
19-45y	Male	19,353	5.6[5.3, 6.4]	5.5[5.4, 5.9]	6.0[5.6, 7.3]	5.7[5.4, 6.5]	+ 0.4
19-45y	Female	15,406	5.4[5.2, 5.8]	5.4[5.2, 5.6]	5.6[5.4, −5.9]	5.5[5.3, 5.8]	+ 0.2
46-60y	Male	45,482	6.0[5.6, 7.3]	5.9[5.5, 6.9]	6.4[5.9, −7.6]	6.3[5.8, 7.5]	+ 0.4
46-60y	Female	40,904	5.8[5.4, 6.4]	5.8[5.5, 6.3]	6.2[5.8, 6.9]	6.1[5.7, 6.9]	+ 0.4
61-75y	Male	47,538	6.1[5.6, 7.1]	6.2[5.7, 7.1]	6.6[6.1, 7.7]	6.5[5.9, 7.5]	+ 0.5
61-75y	Female	46,460	6.0[5.6, 6.9]	6.1[5.7, 6.8]	6.5[6.0, 7.3]	6.4[5.9, 7.3]	+ 0.5
≥76y	Male	29,012	6.0[5.6, 6.9]	6.2[5.8, 7.1]	6.6[6.1, 7.7]	6.5[6.0, 7.5]	+ 0.6
≥76y	Female	30,185	6.0[5.6, 6.8]	6.2[5.8, 6.9]	6.5[6.0, 7.4]	6.5[5.9, 7.4]	+ 0.5

Note. IQR = interquartile range;*:Pre-Pandemic vs Peak Infection period.

#### 3.5.1 Persistent gender disparity

A consistent gender disparity was evident across all age strata. Male patients demonstrated greater susceptibility to glycemic control deterioration compared to their female counterparts within each age category.(Pre-Pandemic: 6.3 % vs 6.0 %; Peak: 6.5 % vs 6.3 %; Post-Pandemic: 6.3 % vs 6.1 %; all *p* < 0.01).

#### 3.5.2 Greatest impact on elderly patients

A pronounced age-dependent gradient was observed in pandemic-related glycemic deterioration. The magnitude of HbA1c elevation progressively increased with advancing age, ranging from a minimal change of +0.1 % in the 0–18 years cohort to the most substantial increase of +0.6 % among patients aged ≥76 years.

### 3.6 Variation in pandemic-related glycemic deterioration across clinical departments

Mann-Whitney *U* tests revealed statistically significant differences between pre-pandemic and peak infection periods for all departments (adjusted *p* < 0.05). Effect sizes (r) varied considerably (Table 4). Critical care departments showed the most pronounced changes. The ICU exhibited the largest effect size (*r* = 0.62, large effect), with median HbA1c rising from 5.8 % to 8.8 %. Emergency Medicine also showed a medium effect size (*r* = 0.37; median HbA1c: 5.8 % to 6.5 %). The Surgical Department demonstrated a medium effect size (*r* = 0.41; median HbA1c: 5.8 % to 6.5 %). Among chronic disease management departments, Neurology showed the largest effect (*r* = 0.49, medium effect; median HbA1c: 5.8 % to 6.5 %), followed by Nephrology (*r* = 0.26), Cardiology (*r* = 0.23), and Health Management (r = 0.23), all with small but significant effects. Notably, the Endocrinology department maintained better glycemic control overall (median HbA1c decreased from 7.1 % to 6.8 %; *r* = 0.15).

**Table 4:** :HbA1c levels by clinical department and pandemic phase among patients at a tertiary hospital in Chengdu, China (2013–2025) (Median[IQR]).

Department	N	Pre-Pandemic	Pandemic	Peak Infection period	Post-Pandemic	MeanDifference*
Endocrinology	41,766	7.1 [6.0, 9.5]	6.8 [6.0, 8.6]	6.8 [6.2, 8.0]	6.9 [6.1, 8.6]	- 0.3
Cardiology	33,160	5.9 [5.6, 6.6]	6.0 [5.6, 6.6]	6.2 [5.8, 6.9]	6.2 [5.8, 6.8]	0.3
Neurology	31,692	5.8 [5.5, 6.3]	5.9 [5.6, 6.5]	6.5 [6.0, 7.8]	6.2 [5.8, 7.1]	0.7
Nephrology	7540	6.2 [5.6, 7.1]	6.3 [5.6, 7.2]	6.8 [6.0, 7.8]	6.5 [5.9, 7.5]	0.6
Emergency Medicine	907	5.8 [5.5, 6.4]	6.5 [5.8, 7.7]	6.5 [5.9, 7.4]	6.2 [5.7, 7.3]	0.7
ICU	1780	5.8 [5.4, 6.7]	6.8 [5.9, 8.6]	8.8 [6.2, 10.8]	6.6 [5.9, 8.2]	3.0
Health Management	54,264	5.7 [5.4, 6.0]	5.6 [5.4, 5.9]	5.9 [5.6, 6.3]	5.7 [5.4, 6.1]	0.2
Surgical Department	26,520	5.8 [5.4, 6.4]	6.1 [5.6, 7.3]	6.5 [5.9, 7.8]	6.5 [5.9, 7.7]	0.7

Note. IQR = interquartile range;*:Pre-Pandemic vs Peak Infection period.

## 4 Discussion

This large cohort study provides a detailed assessment of the COVID-19 pandemic's impact on glycemic control across different phases of the pandemic response in China. We document four key findings: (1) a significant and persistent deterioration in glycemic control that extended into the post-pandemic period; (2) substantial heterogeneity across healthcare delivery settings, with outpatient clinics exhibiting the poorest control; (3) identified vulnerabilities among older adults and male patients; and (4) pronounced disparities across clinical departments, with acute and critical care settings experiencing the greatest disruptions, while specialized diabetes care (Endocrinology) demonstrated resilience.

A critical finding emerging from our analysis is the significantly poorer glycemic control observed in outpatient settings compared to other care settings.The significantly poorer glycemic control observed in outpatient settings compared to inpatient, emergency, and screening visits is a critical finding. This suggests that the pandemic disproportionately disrupted the ongoing, coordinated management of chronic diseases in ambulatory care. While inpatient protocols may have remained intact, and screening programs persisted effectively (Dungan et al., 2009), routine outpatient care likely suffered from reduced follow-up frequency, fragmented coordination, and potentially decreased treatment intensification. This highlights a crucial vulnerability in the healthcare system's backbone for chronic disease management, underscoring the need to bolster outpatient care resilience through integrated care models and digital health solutions. This systemic flaw necessitates that clinicians place greater emphasis on the long-term stability of patient glycemic control by optimizing treatment strategies and enhancing adherence to minimize fluctuations, thereby reducing the risk of diabetic complications (Ceriello and Prattichizzo, 2021).

Beyond highlighting specific care-setting vulnerabilities, the observed overall rise in post-pandemic HbA1c necessitates careful interpretation to distinguish between true deterioration and shifts in the patient population.The observed rise in HbA1c following the pandemic could theoretically be driven by two factors: a genuine deterioration in glycemic control among existing patients, or an influx of newly diagnosed patients with higher baseline HbA1c (a shift in patient mix). While the absence of unique patient identifiers precludes a direct statistical disaggregation of these effects, several observations favor the interpretation of a true negative impact on diabetes management. The persistence of elevated HbA1c levels well into the post-pandemic period argues against a one-time shift in patient demographics and points to sustained systemic challenges. Furthermore, the pronounced heterogeneity across departments is highly informative. The severe deterioration in acute care settings like the ICU and Surgery is most parsimoniously explained by pandemic-related acute stress and care disruptions affecting existing patients. Conversely, the stability within the Endocrinology department demonstrates that dedicated diabetes care was protective. Finally, a potential increase in new, poorly controlled patients presenting to outpatient clinics is, in itself, a significant public health finding, indicating a failure of early detection and management during the pandemic. Therefore, we interpret the overall HbA1c elevation as likely reflecting both a genuine worsening of control in a substantial portion of the population and a system-level delay in diagnosing and engaging new patients.

The pronounced disparities in glycemic outcomes across clinical departments, noted in our key findings, further illuminate the critical role of care specialization. Notably,the superior glycemic outcomes maintained by the Endocrinology department, despite managing complex cases, underscore the value of specialization and expertise in diabetes care (Davies et al., 2022). Conversely, the significant deteriorations observed in departments like Cardiology, Nephrology, and Neurology suggest that diabetes management may be deprioritized when competing with other acute specialist concerns. This is particularly concerning given the importance of glycemic control for preventing complications in these high-risk populations (Harding et al., 2019). Building on this contrast, our results deliver a clear and actionable message: they serve as a powerful argument for implementing and promoting structured shared-care models. We strongly recommend that healthcare institutions develop formal consultation pathways and embedded co-management protocols between endocrinologists and other specialists. Such systems would ensure that diabetes is actively managed as a key comorbidity in all patient settings, leveraging endocrine expertise while allowing other specialties to focus on their primary domain. Optimizing diabetes management through interdisciplinary collaboration is not just beneficial—it is essential for mitigating the long-term complications of diabetes and building a more resilient healthcare system.

In addition to systemic and care-setting factors, our findings reveal important demographic gradients in pandemic-related glycemic vulnerability.The observed age gradient, with the oldest adults (≥76 years) experiencing the greatest magnitude of HbA1c increase, aligns with expectations of higher clinical vulnerability. The persistent gender disparity, however, warrants a life-course perspective. The finding that male patients of productive age exhibited greater susceptibility, while disparities minimized in the young and the old, strongly suggests that sociobehavioral factors, rather than purely biological ones, are the primary drivers of this difference (Kautzky-Willer et al., 2016). We hypothesize that during the productive years, societal gender norms and roles exert a powerful influence on health outcomes. Men are often subject to greater pressures as primary income earners, which may manifest as heightened work-related stress, poorer help-seeking behaviors for chronic conditions, and higher rates of risk-conferring lifestyles (Kautzky-Willer et al., 2016). The COVID-19 pandemic, with its associated economic instability and social disruption, likely acted as a significant amplifier of these pre-existing sociobehavioral vulnerabilities, leading to a more pronounced deterioration in glycemic control among working-age men (Tai et al., 2021). Conversely, the comparable vulnerability seen in children and the very old aligns with a diminishment of these specific societal pressures. The health behaviors of children are largely mediated by their parents or guardians, neutralizing gender-based behavioral differences. Similarly, among older adults aged ≥76 years, retirement and a convergence of social roles may lessen the behavioral gap between genders, allowing biological factors or comorbidity burdens to become the more dominant determinants of glycemic control. This analysis underscores that public health interventions aimed at mitigating gender disparities in chronic disease management must be context-specific. For productive-age males, strategies should focus on reducing stigma around chronic illness, promoting proactive health engagement in workplace settings, and addressing stress and lifestyle factors.

 Our results suggest several actionable recommendations:

(1) **Strengthen Outpatient Care:**Implement strategies to close the outpatient management gap, such as integrating telehealth, remote monitoring, and structured follow-up.
(2) **Promote Shared Care:**Develop formal consultation pathways and shared-care protocols between endocrinology and other specialties (e.g., cardiology, nephrology).
(3) **Target Vulnerable Groups:**Create tailored support programs for older adults and explore interventions addressing the specific needs of male patients to mitigate the observed gender disparity.
(4) **Leverage Successful Models**:Learn from the resilience of screening programs and specialized endocrine care to strengthen overall system response.

The findings of this study should be interpreted in the context of several limitations. First, the single-center design may affect generalizability, though our large sample size provides robust internal validity. Second, we lacked data on diabetes duration, treatment regimens, and socioeconomic factors that might explain some observed disparities. Third, the retrospective and de-identified nature of our dataset meant we could not track individual patients over time. This prevented us from distinguishing between newly diagnosed and returning patients, and thus from fully disentangling the contribution of a shift in patient case-mix from a true deterioration in glycemic control among existing patients. However, the persistence of the effect and the logical patterns observed across clinical settings provide compelling indirect evidence for a genuine pandemic-related impact. Finally, our data come from a tertiary hospital, which may not represent patterns in primary care settings.

## 5 Conclusion

The COVID-19 pandemic had a layered and persistent negative impact on glycemic control, revealing and exacerbating existing disparities within the healthcare system. The recovery of diabetes care requires moving beyond simply restoring pre-pandemic services. It necessitates targeted, differentiated strategies that address the identified vulnerabilities in outpatient care, support non-specialist departments through shared-care models, and protect vulnerable patient groups, such as older adults and male patients, to build a more resilient and equitable system for diabetes management.

Table 2:HbA1c levels by healthcare setting and pandemic phase among patients at a tertiary hospital in Chengdu, China (2013–2025)(Median[IQR]).

**Table:** 

Healthcare Settings	N	Pre-Pandemic	Pandemic	Peak infection period	Post-Pandemic	Overall
Inpatient	162,541	5.9[5.5, 6.8]	6.1[5.6, 6.9]	6.4[5.9, 7.5]	6.5[5.9, 7.7]	6.0[5.6, 7.0]
Outpatient	57,197	6.6[5.9, 7.8]	6.3[5.7, 7.4]	6.5[5.9, 7.5]	6.5[5.8, 7.5]	6.4[5.8, 7.5]
Screening	54,264	5.7[5.4, 6.0]	5.6[5.4, 5.9]	5.9[5.6, 6.3]	5.7[5.4, 6.1]	5.6[5.4, 6.0]
Emergency	907	5.8[5.5, 6.4]	6.5[5.8, 7.7]	6.5[5.9, 7.4]	6.2[5.7, 7.3]	6.1[5.6, 7.0]
Total	274,909	5.9[5.5, 6.8]	5.9[5.5, 6.6]	6.4[5.9, 7.4]	6.2[5.7, 7.2]	6.0[5.6, 6.9]

Note. IQR = interquartile range.

## CRediT authorship contribution statement

**Dongmei Chen:** Writing – review & editing, Writing – original draft, Methodology, Conceptualization. **Shiyu Deng:** Visualization, Formal analysis, Data curation.

## Declaration of competing interest

The authors declare that they have no known competing financial interests or personal relationships that could have appeared to influence the work reported in this paper.

## Data Availability

Data will be made available on request.
